# The “C3aR Antagonist” SB290157 is a Partial C5aR2 Agonist

**DOI:** 10.3389/fphar.2020.591398

**Published:** 2021-01-21

**Authors:** Xaria X. Li, Vinod Kumar, Richard J. Clark, John D. Lee, Trent M. Woodruff

**Affiliations:** School of Biomedical Sciences, The University of Queensland, Brisbane, QLD, Australia

**Keywords:** complement C3a, C3aR, SB290157, C5aR1, C5aR2, complement, C5a anaphylatoxin, C3a anaphylatoxin

## Abstract

Innate immune complement activation generates the C3 and C5 protein cleavage products C3a and C5a, defined classically as anaphylatoxins. C3a activates C3aR, while C5a activates two receptors (C5aR1 and C5aR2) to exert their immunomodulatory activities. The non-peptide compound, SB290157, was originally reported in 2001 as the first C3aR antagonist. In 2005, the first report on the non-selective nature of SB290157 was published, where the compound exerted clear agonistic, not antagonistic, activity in variety of cells. Other studies also documented the non-selective activities of this drug in vivo. These findings severely hamper data interpretation regarding C3aR when using this compound. Unfortunately, given the dearth of C3aR inhibitors, SB290157 still remains widely used to explore C3aR biology (>70 publications to date). Given these issues, in the present study we aimed to further explore SB290157's pharmacological selectivity by screening the drug against three human anaphylatoxin receptors, C3aR, C5aR1 and C5aR2, using cell models. We identified that SB290157 exerts partial agonist activity at C5aR2 by mediating *β*-arrestin recruitment at higher compound doses. This translated to a functional outcome in both human and mouse primary macrophages, where SB290157 significantly dampened C5a-induced ERK signaling. We also confirmed that SB290157 acts as a potent agonist at human C3aR in transfected cells, but as an antagonist in primary human macrophages. Our results therefore provide even more caution against using SB290157 as a research tool to explore C3aR function. Given the reported immunomodulatory and anti-inflammatory activities of C5aR2 agonism, any function observed with SB290157 could be due to these off-target activities.

## Introduction

The complement system is an essential component of innate immunity, responding to external and internal insults. Complement activation, through all pathways including the classical, lectin and alternative pathways, generates the cleavage complement peptides C3a and C5a, which serve as important innate effectors, modulating multiple aspects of immune cell function (Ricklin et al., 2016). C3a is one of the most abundant complement activation products generated from the cleavage of C3 by C3 convertases (Coulthard and Woodruff, 2015). Human C3a, consisting of 77 amino acids (Klos et al., 2009), interacts potently with the canonical 7-transmembrane G-protein coupled receptor, C3a receptor (C3aR), which is widely expressed by all myeloid cells (such as neutrophils and monocytes), activated T lymphocytes and B lymphocytes (Fischer and Hugli, 1997; Martin et al., 1997; Zwirner et al., 1999; Werfel et al., 2000; Klos et al., 2013; Wu et al., 2013), and also non-leukocytes such as neuronal progenitors, microglia, astrocytes and endothelial cells (Klos et al., 2013; Coulthard et al., 2018). C3aR is predominantly coupled to the pertussis toxin-sensitive G protein G*α*_i2_ (Norgauer et al., 1993). Upon activation, C3aR dampens the intracellular cAMP/PKA signaling, induces the PI3K/Akt pathway, intracellular calcium mobilisation and extracellular signal-regulated kinase 1/2 (ERK1/2) phosphorylation, and recruits *β*-arrestins (Klos et al., 2013). The nature of C3a action appears to be rather nuanced, with both pro- and anti-inflammatory “opposing” responses reported in the literature (Proctor et al., 2009; Coulthard and Woodruff, 2015).

The non-peptide small molecule SB290157 was first reported in 2001 (Ames et al., 2001). Functioning as a competitive C3aR antagonist (Ames et al., 2001), SB290157 has served as a major pharmacological tool used to explore C3aR biology. For instance, SB290157 treatment was shown to improve survival in experimental lupus nephritis (Bao et al., 2005), ameliorate anti-ovalbumin polyclonal antibody–induced arthritis (Hutamekalin et al., 2010), reduce infarct volume in a model of thromboembolic stroke (Ahmad et al., 2020), and rescue cognitive impairment in a model of Alzheimer's disease (Lian et al., 2015). The promising results shown by administering SB290157 in these experimental models has heightened interest in C3aR blockade as a therapeutic strategy for diverse diseases. However, despite the promising initial preclinical efficacy of SB290157, studies performed in the mid 2000's demonstrated that this compound possessed clear agonistic (not antagonistic) activities on C3aR, in cells with high levels of C3aR expression (Mathieu et al., 2005). Furthermore, independent studies document undefined off-target effects when SB290157 was administered *in vivo*, even at relatively low doses (Proctor et al., 2004; Woodruff and Tenner, 2015). Likely due to these non-selective activities, SB290157 has not progressed commercially as a therapeutic. However, despite these on- and off-target limitations, SB290157 continues to be used widely in the field to block C3aR activity (Lee et al., 2020). Indeed, to the best of our knowledge, over 70 research articles have used this drug to explore roles for C3aR, presumably in part due to the limited availability of alternate therapeutic approaches.

Human C3aR shares 38 % (116/309 residues) similarity with human C5aR1 (Crass et al., 1996; Klos et al., 2013), and a 55% similarity in the transmembrane domains with C5aR2 (Lee et al., 2001). Due to the close homology between C3a and C5a receptors, ligands designed to target one of these receptors may have promiscuous actions on the other two (Halai et al., 2014; Pandey et al., 2019). SB290157 was previously shown to be devoid of C5aR1-antagonistic properties in human neutrophils or C5aR1-expressing rat basophilic leukemia cells (Ames et al., 2001). However, to date, whether SB290157 exerts activity on C5aR2 has not been reported. In the present study, we thus aimed to further characterise the pharmacological activity of SB290157 on all three anaphylatoxin receptors, C3aR, C5aR1 and C5aR2, by employing various signaling assays in transfected cell lines and primary human and mouse macrophages. We identified that in addition to acting as a C3aR agonist on transfected cells, SB290157 was also a partial agonist for C5aR2, which conferred a functional consequence by down-regulating C5a-induced ERK signaling in primary human and mouse macrophages. This study thus further highlights caution against utilising this drug in experimental studies examining C3aR biology.

## Materials and Methods

### Ligands and Materials

The C3aR inhibitor, SB290157 trifluoroacetate salt, and purified human C3a was purchased from Merck (Perth, Australia). The C5aR2 selective ligand, P32, was synthesized in house as previously described (Li et al., 2020a). Recombinant human C5a and recombinant mouse C5a were purchased from Sino Biological (Beijing, China). Bovine serum albumin (BSA) was purchased from Merck (Perth, Australia). For cell culture, trypsin-EDTA, HBSS, HEPES, Dulbecco's Modified Eagle's Medium (DMEM), phenol-red free DMEM, Ham's F12, Iscove's Modified Dulbecco's Medium, RPMI-1640 and Penicillin-Streptomycin were purchased from Thermo Fisher Scientific (Melbourne, Australia). Dulbecco's phosphate-buffered saline was purchased from Lonza (Melbourne, Australia). Stocks of both ligands, SB290157 and P32, were reconstituted in DMSO, at 10 and 100 mM, respectively.

### Cell Culture

The following cell lines were cultured as previously described (Croker et al., 2013). Briefly, Chinese hamster ovary cells stably expressing the human C5aR1 (CHO-C5aR1) or human C3aR (CHO-C3aR) were maintained in Ham's F12 medium containing 10% fetal bovine serum (FBS), 100 IU/ml penicillin, 100 μg/ml streptomycin and 400 μg/ml G418 (InvivoGen, San Diego, United States). Human embryonic kidney 293 cells (HEK293) were maintained in DMEM medium containing 10% FBS, 100 IU/ml penicillin and 100 μg/ml streptomycin. All cell lines were maintained in T175 flasks (37°C, 5% CO_2_) and subcultured at 80–90% confluency using 0.05% trypsin-EDTA in DPBS.

Human monocyte-derived macrophages (HMDMs) were generated and cultured as previously described (Li et al., 2020a; Li et al., 2020c). Briefly, human buffy coat blood from anonymous healthy donors was obtained through the Australian Red Cross Blood Service (Brisbane, Australia). Human CD14^+^ monocytes were isolated from blood using Lymphoprep density centrifugation (STEMCELL, Melbourne, Australia) followed by CD14^+^ MACS magnetic bead separation (Miltenyi Biotec, Sydney, Australia). The isolated monocytes were differentiated for 7 days in Iscove’s Modified Dulbecco’s Medium supplemented with 10% FBS, 100 IU/ml penicillin, 100 μg/ml streptomycin and 15 ng/ml recombinant human macrophage colony stimulating factor (Lonza, Melbourne, Australia) on 10 mm square dishes (Bio-strategy, Brisbane, Australia). Non-adherent cells were removed by washing with DPBS, and the adherent differentiated HMDMs were harvested by gentle scraping.

Mouse bone marrow-derived macrophages (BMDMs) were obtained and cultured as previously described (Davies and Gordon, 2005). Briefly, mice were anesthetised and then sacrificed by cervical dislocation. The tibia were removed and sterilised. Upon removal of both epiphyses, bone marrow cells were harvested by flushing the central cavity using a 10 ml syringe attached to a 25-gauge needle. Cells were then cultured in complete RPMI-1640 medium (containing 10% FBS, 100 IU/ml penicillin, 100 μg/ml streptomycin) supplemented with 100 ng/ml recombinant human macrophage colony stimulating factor on 10 mm square Petri dishes. Mature adherent macrophages for assays were harvested on day 6–7 by gentle scraping.

### Phospho-Extracellular Signal-Regulated Kinase 1/2 Assays

Ligand-induced ERK1/2 phosphorylation was assessed using the AlphaLISA *Surefire Ultra p*-ERK1/2 (Thr202/Tyr204) kit (PerkinElmer, Melbourne, Australia) following the manufacturer's protocol as previously described (Li et al., 2020a; Li et al., 2020c). Briefly, CHO-C3aR, CHO-C5aR1, HMDMs (50,000/well) and BMDMs (90,000/well) were seeded in tissue culture-treated 96-well plates (Corning, United States) for 24 h and serum-starved overnight. All ligand dilutions were prepared in serum-free medium (SFM) containing 0.1% BSA. For inhibition assays, cells were pre-treated with the indicated ligands or the solvent-only control (DMSO) for 30 min before agonist addition. For stimulation, cells were treated with ligands at the indicated concentrations for 10 min at room temperature and then immediately lysed using AlphaLISA lysis buffer on a microplate shaker (450 rpm, 10 min). For the detection of phospho-ERK1/2 content, cell lysate (5 μL/well) was transferred to a 384-well ProxiPlate (PerkinElmer, Melbourne, Australia) and added to the donor and acceptor reaction mix (2.5 μL/well, respectively), followed by a 2-h incubation at room temperature in the dark. On a Tecan Spark 20 M (Tecan, Männedorf, Switzerland), the plate was measured using standard AlphaLISA settings.

### Bioluminescence Resonance Energy Transfer Assays Measuring *β*-arrestin 2 Recruitment to C5aR2

The C5a-mediated *β*-arrestin 2 recruitment to C5aR2 was measured using bioluminescence resonance energy transfer (BRET)-based assay as previously described (Croker et al., 2014; Li et al., 2020c). Briefly, HEK293 cells were transiently transfected with *β*-arrestin 2-*Renilla* luciferase 8 (Rluc8) and human C5aR2-Venus constructs using XTG9 (Roche, Sydney, Australia) for 24 h. Transfected cells were then seeded (100,000/well) onto white 96-well plates (Corning, United States) in phenol-red free DMEM containing 5% FBS overnight. For BRET assay, cells were firstly incubated with the substrate EnduRen (30 μM, Promega, Sydney, Australia) for 2 h (37°C, 5% CO_2_). All ligands were prepared in SFM containing 0.1% BSA. On a Tecan Spark 20 M microplate reader (Tecan, Männedorf, Switzerland) (37°C), the BRET light emissions (460–485 and 520–545 nm) were continuously monitored for 25 reads with respective ligands added after the first 5 reads. The ligand-induced BRET ratio was calculated by subtracting the emission ratio of Venus (520–545 nm)/Rluc8 (460–485 nm) of the vehicle-treated wells from that of the ligand-treated wells.

### Data Collection, Processing and Analysis

All experiments were conducted in triplicates and repeated on 3 separate days (for cell lines), using cells from 3 or more independent donors (for HMDMs) or mice (for BMDMs) unless otherwise specified. Data was analysed using GraphPad software (Prism 8.4). Data from each individual repeat was normalised accordingly before being combined and expressed as mean ± standard error of the mean (S.E.M.) unless otherwise described. For all dose-response studies, logarithmic concentration-response curves were plotted using combined data and analysed to determine the respective potency values. Statistical analysis was performed using unpaired Student's *t*-test or two-way ANOVA as indicated. Differences were deemed significant when *p* < 0.05.

## Results

### SB290157 Potently Activates Extracellular Signal-Regulated Kinase Signaling in Chinese Hamster Ovary Cells Stably Expressing C3aR

**FIGURE 1:**
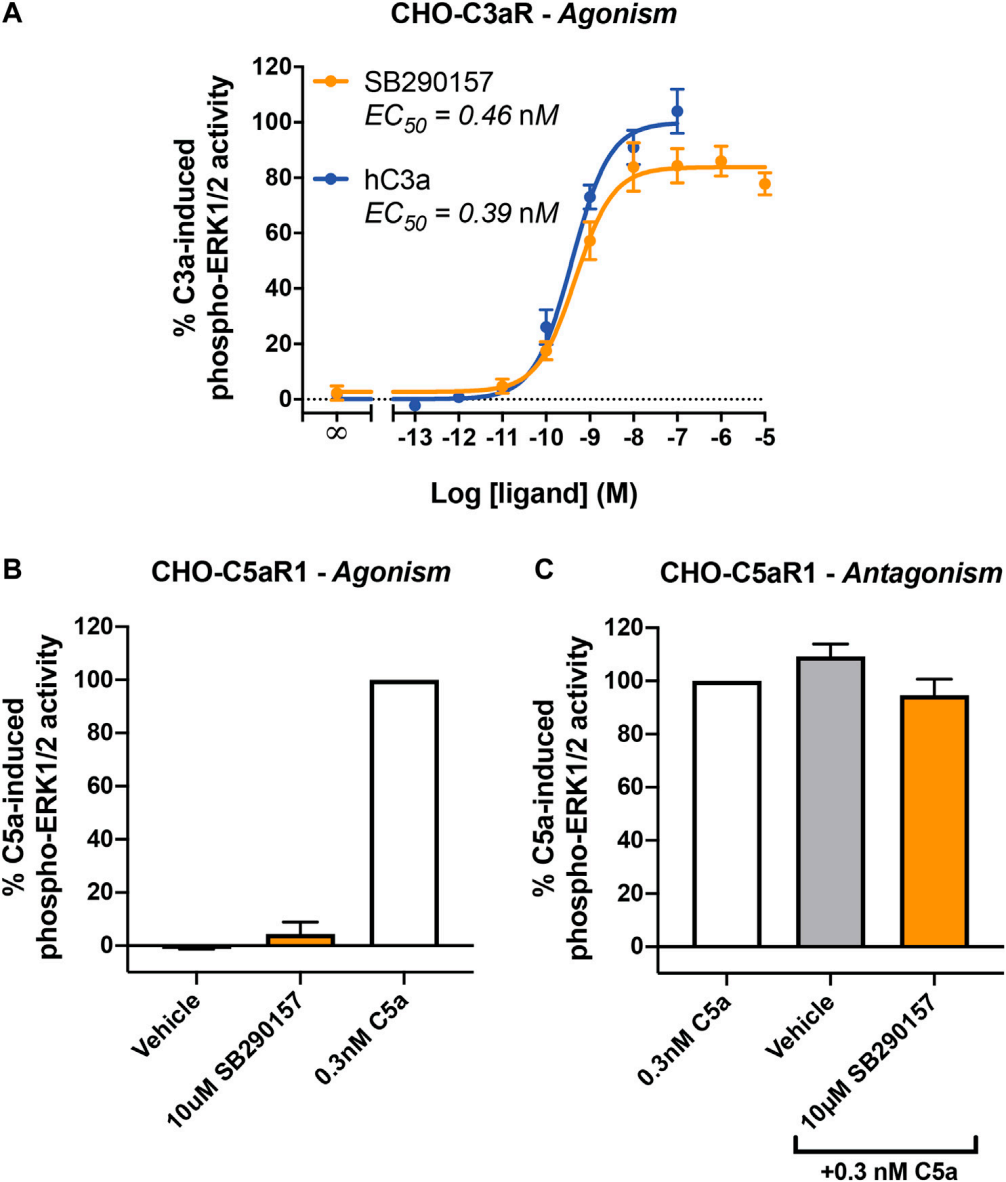
SB290157 potently activates C3aR-mediated ERK signaling in transfected CHO cells. **(A)** Agonism testing of SB290157 on CHO-C3aR cells. Serum-starved CHO-C3aR cells (50,000/well) were stimulated with respective concentrations of SB290157 or purified human C3a for 10 min before being lysed. **(B)** Agonism testing of SB290157 on CHO-C5aR1 cells. Serum-starved CHO-C5aR1 cells (50,000/well) were stimulated with the respective ligands at the indicated concentrations for 10 min before being lysed. **(C)** Antagonism testing of SB290157 on CHO-C5aR1 cells. Serum-starved CHO-C5aR1 cells (50,000/well) were pre-treated with SB290157 (10 µM) or vehicle (0.1% DMSO) for 30 min before being stimulated with 0.3 nM of C5a for 10 min and then lysed. The phospho-ERK1/2 content in the cell lysate was measured and normalised to the maximum C3a-induced (for **A**) or 0.3 nM C5a-induced (for **B**, **C**) levels before being combined. Data represent mean ± S.E.M. of triplicate measurements from 3 to 6 independent experiments (n = 3–6).

First, we aimed to confirm the prior reported C3aR-agonistic property of SB290157 on an overexpression cell line (Mathieu et al., 2005). We measured C3aR-mediated ERK signaling as a readout in CHO cells stably expressing human C3aR. As expected, SB290157 potently induced phosphorylation of ERK1/2 in CHO-C3aR cells in a dose-dependent fashion, with a highly potent EC_50_ of 0.46 nM (Figure 1A). Notably, the sub-nanomolar potency of SB290157 was comparable to the 0.39 nM potency demonstrated by human C3a, however, the maximum level of C3aR activation caused by SB290157 was 16% lower than human C3a (*p* = 0.0082, unpaired Student's t-test), similar to a prior report (Mathieu et al., 2005). When SB290157 was counter-screened in CHO cells overexpressing human C5aR1, the ligand did not cause significant activation or inhibition of human C5aR1-mediated phospho-ERK1/2 activity (Figures 1B,C). Therefore, we confirmed that SB290157 behaved as potent agonist in C3aR-transfected cells, but was inactive at C5aR1.

### SB290157 Possesses Off-Target Activity on Human C5aR2

We next examined the potential activity of SB290157 on the other closely related complement receptor, C5aR2. As a non-canonical G-protein coupled receptor, C5aR2 does not couple to the common classes of G proteins and is devoid of the classical G protein-mediated signaling activities (Li et al., 2019; Pandey et al., 2020). Human C5aR2 activation however recruits *β*-arrestins, which can be used as a readout of receptor activation (Bamberg et al., 2010; Croker et al., 2014). We thus assessed the potential activity of SB290157 on human C5aR2-β-arrestin 2 interaction using a BRET assay established in HEK293 cells, and compared to that of the existing and widely used C5aR2-selective agonist, P32 (Ac-RHYPYWR-OH) (Croker et al., 2016).

**FIGURE 2:**
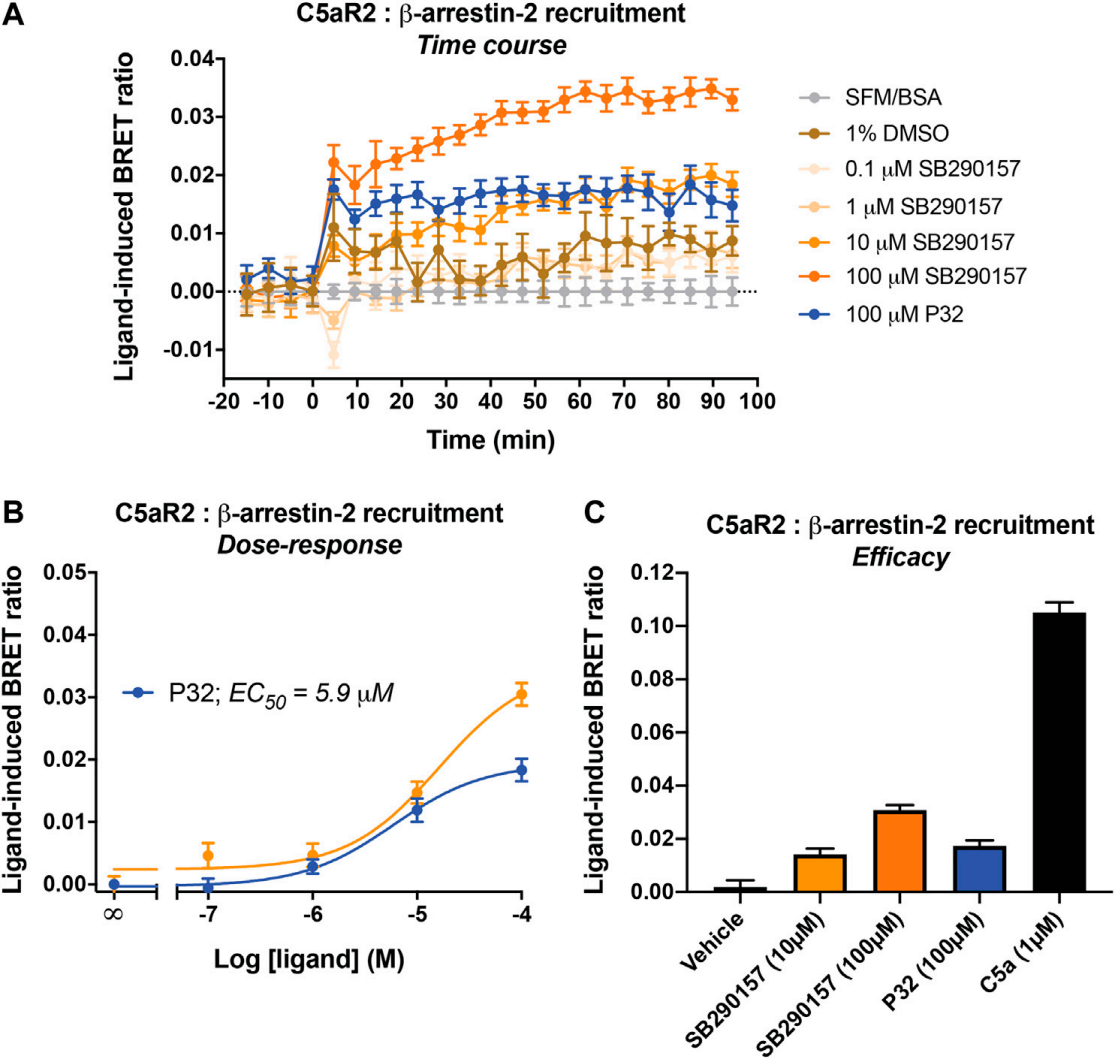
SB290157 induces C5aR2-mediated *β*-arrestin 2 recruitment in transfected HEK293 cells. HEK293 cells were transiently transfected using C5aR2-Venus and *β*-arrestin 2-Rluc8 BRET pairs for 24 h and seeded (100,000/well) overnight. Filtered light emissions between 460–485 nm (Rluc8) and 520–545 nm (Venus) were continually monitored for 90 min with SB290157, P32, C5a or vehicle added at the 0 min time point. Data represent **(A)** the time course of ligand-induced BRET ratios (Venus/Rluc8 emission ratio) caused by the respective ligands at the indicated concentrations, **(B)** the corresponding concentration-response curves for SB290157 and P32 at 40 min post ligand addition, and **(C)** the ligand-induced BRET ratios (efficacy) of SB290157, P32 or C5a at 40 min post ligand addition. Data represent the mean ± S.E.M. of triplicate measurements from 3 to 5 independent experiments (n = 3–5).

Surprisingly, SB290157 dose-dependently induced C5aR2-mediated *β*-arrestin 2 recruitment, with a micromolar potency (EC_50_) of 16.1 µM (Figures 2A,B), which is slightly less potent compared to P32 (EC_50_ = 5.9 µM). The maximum level of *β*-arrestin 2 recruitment to C5aR2 induced by SB290157 however was higher relative to that of P32. We next compared the C5aR2 activity of SB290157 to that of human C5a (Figure 2C), and observed that, similar to P32, the C5aR2-agonistic activity of SB290157 was also partial, reaching 31% of the level induced by C5a. Thus, our results clearly show that at higher concentrations, SB290157 also partially activates C5aR2.

### SB290157 Potently Inhibits C3a-Mediated Extracellular Signal-Regulated Kinase Signaling in Human Monocyte-Derived Macrophages

**FIGURE 3:**
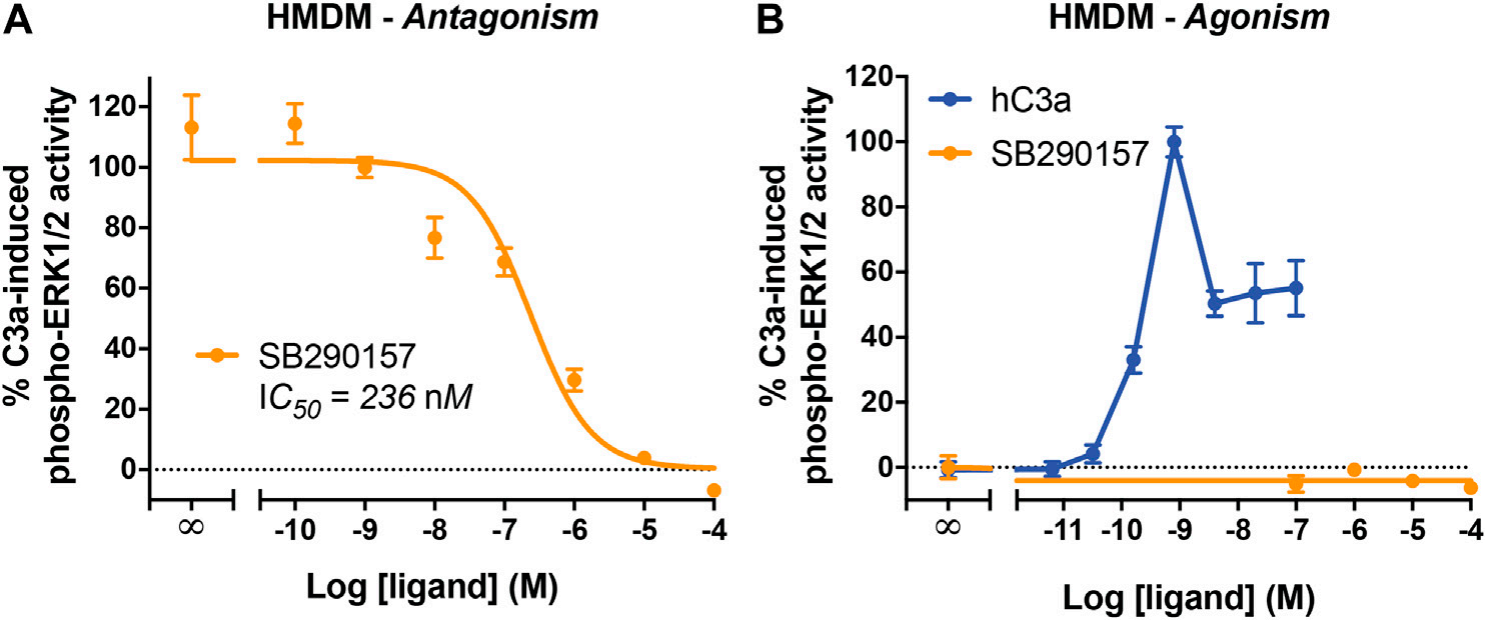
SB290157 potently inhibits C3aR-mediated ERK signaling in human monocyte-derived macrophages. **(A)** Antagonism testing of SB290157 on HMDMs. Serum-starved HMDMs (50,000/well) were pre-treated with various doses of SB290157 for 30 min before being stimulated with purified human C3a (5 nM) for 10 min and then lysed. **(B)** Agonism testing of SB290157 on HMDMs. HMDMs were stimulated with respective doses SB290157 for 10 min and then lysed. The phospho-ERK1/2 content in the cell lysate was measured and normalised to the C3a-induced levels before being combined. Data represent mean ± S.E.M. of triplicate measurements using cells from 2-3 independent donors (n = 2–3).

We next aimed to determine the activity of SB290157 in primary human immune cells, specifically HMDMs, which is an established and widely used model of resting tissue macrophages that express high levels of human C3aR and the C5a receptors (Lacey et al., 2012; Mommert et al., 2018). Human C3a potently activates ERK1/2 phosphorylation in HMDMs (Li et al., 2020a). We therefore assessed the ability of SB290157 to inhibit this C3a-mediated signaling pathway, and found that this compound dose-dependently inhibited C3a-induced ERK signaling in HMDMs (Figure 3A). The IC_50_ of SB290157 (236 nM), was ∼16-fold more potent than previously reported data (IC_50_ = 3.8 µM), determined using an intracellular calcium mobilisation assay in the same cells (Rowley et al., 2020). The discrepancy could be explained by the competitive nature of SB290157-mediated inhibition. The lower C3a concentration used in the present study, 5 vs. 100 nM in Rowley et al. (2020), would give rise to a lower apparent IC_50_ value of SB290157 (Wyllie and Chen, 2007). In marked contrast to the CHO-C3aR data, SB290157 did not display any agonistic activity on ERK signaling in HMDMs at the highest possible concentration tested of 100 μM, although human C3a displayed an EC_50_ of 0.21 nM (Figure 3B), in accordance with the previous data (Li et al., 2020a; Rowley et al., 2020). We also performed dose titrations of SB290157 to ensure the absence of agonism was not a result of the ERK signaling being down-regulated, as experienced by higher concentrations of human C3aR (Figure 3B). Thus, at least for primary human macrophages, SB290157 retains its potent C3aR-inhibitory activity.

### C5aR2 Activation Dampens C5a-Induced Extracellular Signal-Regulated Kinase Signaling in Primary Human and Mouse Macrophages

**FIGURE 4:**
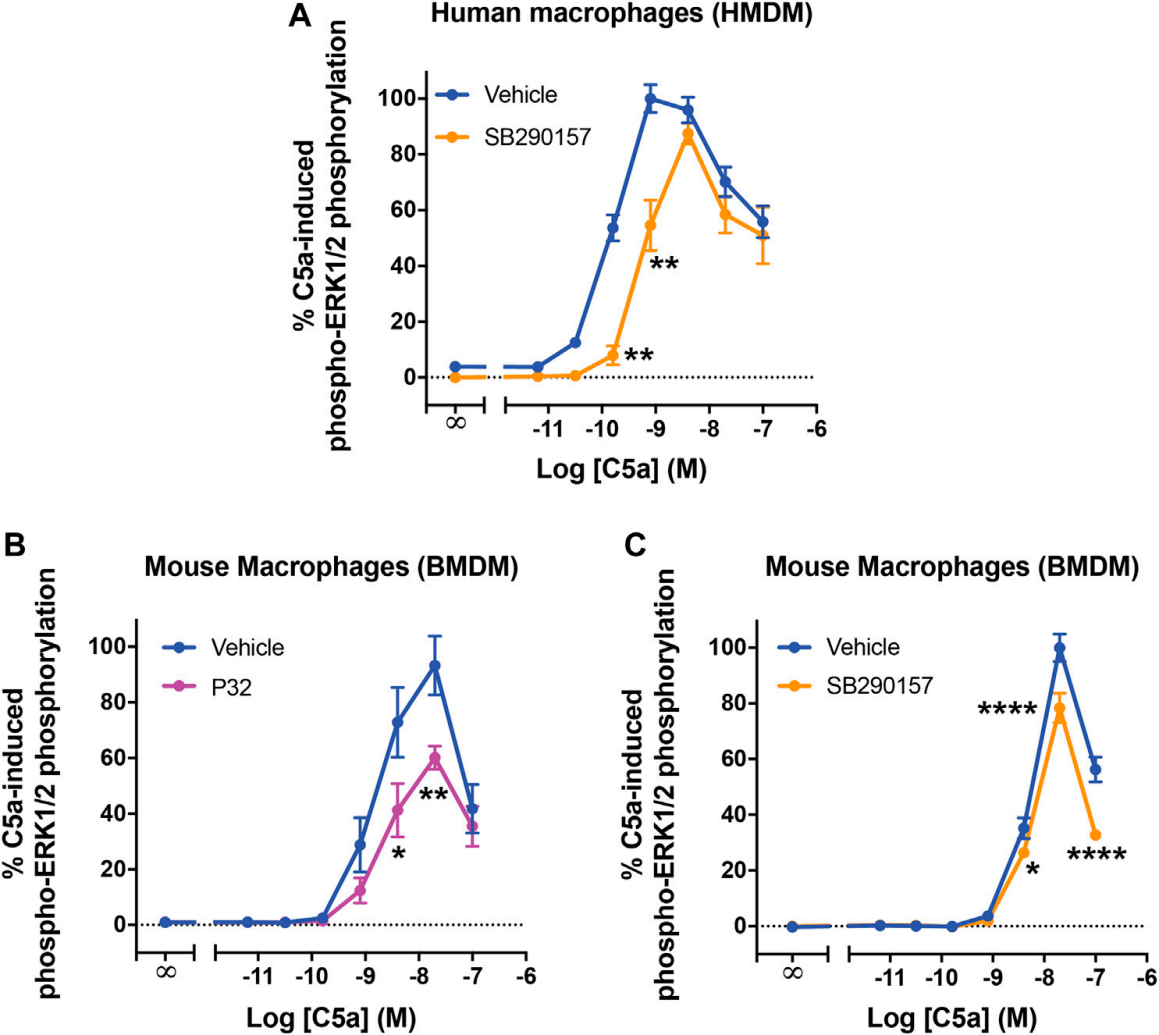
C5aR2 activation dampens C5a-induced ERK signaling in human monocyte-derived macrophages and mouse bone marrow-derive macrophages. HMDMs **(A)** or BMDMs **(B,C)** were serum-starved overnight and pre-treated with SB290157 (50 µM) or P32 (100 µM) and the corresponding solvent-only control (Vehicle) for 30 min, prior to stimulation with recombinant human C5a (10 min, for **A)** or recombinant mouse C5a (5 min, for **B and C)**. The phospho-ERK1/2 content in the cell lysate was measured and normalised to the maximum vehicle-treated C5a-induced levels before being combined. Data represent mean ± S.E.M. of triplicate measurements using cells from 3-6 independent donors (n = 3–6) for HMDMs, or 4 mice (n = 4) for BMDMs. Two-way ANOVA with Sidak's post hoc test. *p < 0.05, **p < 0.01, ****p < 0.0001, P32 or SB290157 pre-treated vs. control-treated cells stimulated by respective concentrations of C5a.

We next evaluated the functional relevance of the SB290157-induced C5aR2 activation in primary macrophages. Previous studies have shown that C5aR2 activation significantly dampens C5a-induced ERK signaling in primary human macrophages and polymorphonuclear leukocytes (Bamberg et al., 2010; Croker et al., 2014; Croker et al., 2016). In particular, pre-treating HMDMs with the C5aR2-selective agonist P32 significantly shifted the concentration-response curve of C5a, with a mild reduction in potency and the peak response (Li et al., 2020a). We thus examined whether pre-treating HMDMs with SB290157 may impact C5a-mediated ERK signaling in a similar fashion. Notably, SB290157 pre-treatment significantly dampened C5a-induced ERK signaling, reducing its EC_50_ potency from 0.16 to 0.62 nM, and the peak response to 87% of the control level (Figure 4A).

Considering the wide usage of SB290157 in murine disease models, we subsequently investigated how C5aR2 activation, and that induced by SB290157, may affect C5a-induced ERK signaling in mouse primary macrophages. Indeed, C5aR2-mediated downregulation of C5a-induced ERK signaling has been reported in mouse primary bone marrow-derived neutrophils (Karsten et al., 2017). We first examined whether this C5aR2-mediated effect was also present in mouse bone marrow-derived macrophages (BMDMs). C5a potently activated ERK signaling in BMDMs (EC_50_ = 1.6 nM) (Figure 4B), with a marked reduction in ERK signaling at a higher dose, showing a comparable trend to that in HMDMs (Figure 4A). Pre-treating cells with the C5aR2-selective agonist P32, significantly reduced the peak activity of C5a to 60% of the control level (Figure 4B). Pre-treating BMDMs with SB290157 also reduced the C5a peak response to 79% of the control measurement (Figure 4C). Therefore, we confirmed that activating C5aR2 using the C5aR2-selective agonist P32, or SB290157, down-regulated C5a-induced ERK signaling in BMDMs.

## Discussion

In this study, we examined the pharmacological properties of the widely-used C3aR antagonist, SB290157 on human C3aR, C5aR1 and C5aR2 receptors, utilising both transfected overexpression cell lines and primary human macrophages. First, in an overexpression system of CHO-C3aR cells, we confirmed the agonistic property of SB290157 on ERK signaling, mediated through C3aR. The sub-nanomolar potency of SB290157 is comparable to that of human C3a, despite SB290157's much lower affinity on human C3aR compared to C3a (K_i_ = 210 nM for SB290157, vs. 0.09 nM for human C3a) (Mathieu et al., 2005). To account for this, the high K_i_ of SB290157 mainly results from its relatively large K_off_ rate (Mathieu et al., 2005). This property is not reflected well by measuring ERK signaling, a pathway characterised by extensive amplification, such that a transient activation of a small proportion of receptors is sufficient to trigger a complete response (Smith et al., 2018). Thus, our results indicate that SB290157 may act as a partial agonist with low intrinsic efficacy, which can elicit a near-complete response in cells with high receptor density (Hanania et al., 2002; Mathieu et al., 2005).

Next, when counter-screening against a closely related complement receptor, C5aR2, we observed a partial, yet significant agonistic effect from SB290157. Albeit being less potent than the existing C5aR2-selective agonist P32 (Croker et al., 2016), SB290157 displayed an improved efficacy at C5aR2. This result indicates that higher doses of SB290157 could activate C5aR2 to modulate immune responses. Indeed, multiple studies have utilised P32 as a pharmacological tool to decipher the roles of C5aR2 (Arbore et al., 2016; Croker et al., 2016; West et al., 2017; Verghese et al., 2018), and we recently documented that P32 exerted profound modulatory effects on multiple signaling and functional responses of HMDMs (Li et al., 2020a). In particular, pre-treating HMDMs with P32 was previously shown to significantly dampen C5a-induced ERK signaling, likely through *β*-arrestin-mediated, yet-to-be-identified mechanisms (Li et al., 2020a), and this down-regulatory effect was also displayed by SB290157, and in fact to a greater extent than P32. The enhanced phospho-ERK inhibitory ability of SB290157 could be explained by its higher efficacy on human C5aR2 relative to P32. The C5aR2-mediated down-regulation of C5a-induced ERK signaling was also recapitulated in mouse macrophages (i.e., BMDMs). although to a lesser extent than that observed in HMDMs. Notably, SB290157 significantly affected phospho-ERK signaling at C5a doses as low as 0.2 nM (human) and 3 nM (mouse). In addition, several C5aR1/2 partial agonists, for instance P32 and C028 (C5a^Pep^, NMe-FKPdChaChadR-OH), exhibit biased agonism among different cell systems and signaling pathways (Croker et al., 2016; Pandey et al., 2019), which also translate to their varied modulatory functions in human primary immune cells (Pandey et al., 2019; Li et al., 2020a). It would thus be interesting to explore in future studies whether the partial activity of SB290157 on human C5aR2 may also lead to biased functional outcomes.

In a previous study by Proctor et al. (2004), SB290157 demonstrated anti-inflammatory activities in a rat model of intestinal ischemia/reperfusion injury, which was associated with ligand-induced global neutrophil tissue sequestration during ischemia, rather than pure C3aR antagonism. C5aR2 is expressed on polymorphonuclear leukocytes, including neutrophils, and has been to shown to suppress C5a-induced neutrophil chemotaxis, both *in vitro* and *in vivo* (Bamberg et al., 2010; Croker et al., 2016). Given the results of our study, it is possible the non-specific neutrophil effect of SB290157 depicted by Proctor et al., (2004) could have resulted from the ligand-induced activation of C5aR2.

It is not uncommon for SB290157 to be used at doses higher than needed to block C3aR. For example, doses of 10–30 mg/kg SB290157 are commonly used *in vivo* (Hutamekalin et al., 2010; Ahmad et al., 2020; Shu et al., 2020), and doses of 10 µM are used *in vitro* (Ahmad et al., 2019; Litvinchuk et al., 2018). Based on prior pharmacokinetic reports as summarized in Table 1, intravenous (*i.v.*) administration of any dose greater than 1 mg/kg could result in a sufficient circulatory concentration of SB290157 to target both C3aR and C5aR2 based on its calculated IC_50_ and EC_50_. Likewise, intraperitoneal (*i.p.*) administration of dose greater than 10 mg/kg could also lead to a circulatory concentration that ultimately targets both receptors. Hence, administering a dose greater than 10 mg/kg (*i.p.*) or 0.3 mg/kg (*i.v.*), would likely result in both agonistic activity at C5aR2 and antagonist/agonist activity at C3aR. High *in vivo* doses of SB290157 could therefore lead to numerous on- and off-target adverse effects such as neutropenia (Proctor et al., 2004), transient hypertension (Proctor et al., 2004), gain of body weight (Hutamekalin et al., 2010), hematopoietic effects (Ratajczak et al., 2004) and tachycardia (Őrfi et al., 2019), that would severely impact on data interpretation. Our data thus further highlights the importance of proper characterisation of drug pharmacokinetics to pharmacological profile when utilising compounds *in vivo* (Woodruff and Tenner, 2015). Considering the broad expression of C5aR2 in immune cells and intricate interplay between C5aR2 and C3aR (Chen et al., 2007; Klos et al., 2013; Li et al., 2020a), the utilisation of high concentrations of SB290157 in primary immune cells, or *in vivo*, should be avoided whenever possible.

**TABLE 1:** Calculated molar concentration of SB290157 after various routes and doses of administration.

Species	Route	Dose (mg kg^−1^)	Cmax	Predicted C_0_	Molar concentration	References
Rat	*i.v*	1	—	>15 μg/ml	>36 µM	Proctor et al. (2004)
		0.3	—	>3 μg/ml	>7 µM	
		0.1	—	>1 μg/ml	>2 µM	
Guinea pig	*i.p*	30	>7 μg/ml	—	>16 µM	Ames et al. (2001), Woodruff and Tenner (2015)
Mice	*i.p*	1; 3 times per week	>0.2 μg/ml	—	>400 nM	Lian et al. (2015), Woodruff and Tenner (2015)

Finally, despite the on- and off-target limitations of SB290157 (i.e., C3aR agonist and C5aR2 agonist activity), in macrophages that naturally express complement-peptide receptors, SB290157 effectively and potently inhibited C3a-induced phospho-ERK1/2 signaling, without any agonistic effect. This confirms prior reports that the pharmacological C3aR agonist activity of SB290157 may be dependent on cellular receptor density (Mathieu et al., 2005). For instance, the level of C3aR expression in HMDMs is estimated to be in the fmol/mg range (Zwirner et al., 1999; Seow, 2014), while that of the CHO-C3aR cell line utilised in the current study is estimated to be 27 ± 7.6 pmol/mg protein based on the manufacturer's data (up to 20,000-times higher than that of HMDMs). The C3aR activation triggered by the weak agonist effects of SB290157 may thus fail to reach the threshold for activating the ERK signaling pathway in the majority of the HMDM population (Birtwistle et al., 2012). In addition, primary cells are known to possess much more intricate regulatory networks in comparison to a simple, overexpression cell line (Rao, 2001; Li et al., 2020c), which could also explain for the absence of C3aR-mediated signaling in the presence of a weak agonist in primary macrophages. As such, extra precaution needs to be taken when interpreting data generated with this ligand in overexpression cell systems, or native cells with naturally high levels of C3aR, such as mast cells (Zwirner et al., 1999), or under situations where there may be an upregulation of C3aR expression (Van Beek et al., 2000; Brennan et al., 2019). It should also be noted that we cannot exclude the possibility that some of SB290157-mediated dampening effects on C3aR-mediated ERK signaling in HMDMs have indirectly resulted from C5aR2 agonism (Li et al., 2020a), especially when high concentrations of SB290157 were used.

In conclusion, our results confirm that the widely used C3aR antagonist SB290157, exerts potent C3a agonist activity in transfected cells, and partial C5aR2 agonist activity at higher concentrations. As many studies continue to use this drug at concentration ranges that would exert activity at C5aR2, it is possible some of the reported phenotypes are due to activity at this receptor, in addition to any potential activity at C3aR. Thus, our data further caution against the use of this compound as the sole tool to delinate C3aR biology and function. Until further validated selective C3aR inhibitory tools become available (eg. Rowley et al., 2020), we strongly recommend the use of other genetic approaches such as gene-knockout mice, or gene-knockdown approaches, to validate roles for C3aR. If SB290157 usage is absolutely required (although we urge against this), an *in vivo* dose of no more than 1 mg/kg (*i.p.*) should be considered to minimise off-target activity at C5aR2.

## Data Availability Statement

The raw data supporting the conclusions of this article will be made available by the authors, without undue reservation.

## Ethics Statement

The studies involving human participants were reviewed and approved by The University of Queensland Human Research Ethics Committee. The patients/participants provided their written informed consent to participate in this study.

## Author Contributions

TW, JL, and XL conceived the project and designed the research; XL performed the research, analysed data and wrote the first paper draft. RC and JL generated key reagents for the study, and VK provided additional pharmacokinetic data analyses and insight. All authors edited the manuscript, and approved the final version.

## Funding

This work was supported by National Health and Medical Research Council of Australia (NHMRC) (Grant APP1118881 to TW and RC).

## Conflict of Interest

The authors declare that the research was conducted in the absence of any commercial or financial relationships that could be construed as a potential conflict of interest.

## Abbreviations

BMDM, bone marrow-derived macrophage; BRET, bioluminescence resonance energy transfer; BSA, bovine serum albumin; C3aR, C3a receptor C5aR1, C5a receptor 1; CHO-C3aR, Chinese hamster ovary cells stably expressing C3aR; CHO-C5aR1, Chinese hamster ovary cells stably expressing C5aR1; DMEM, Dulbecco's Modified Eagle’s Medium; ERK1/2, extracellular signal-regulated kinase 1/2; FBS, fetal bovine serum; HEK293, human embryonic kidney 293 cells; HMDM, human monocyte-derived macrophage; *i.p.*, intraperitoneal; *i.v.*, intravenous; S.E.M. standard error of the mean; SFM, serum-free medium.
